# Comparisons of the Postprandial Inflammatory and Endotoxaemic Responses to Mixed Meals in Young and Older Individuals: A Randomised Trial

**DOI:** 10.3390/nu9040354

**Published:** 2017-04-02

**Authors:** Amber M. Milan, Shikha Pundir, Chantal A. Pileggi, James F. Markworth, Paul A. Lewandowski, David Cameron-Smith

**Affiliations:** 1 Liggins Institute, University of Auckland, Private Bag 92019, Auckland 1023, New Zealand; a.milan@auckland.ac.nz (A.M.M.); s.pundir@auckland.ac.nz (S.P.); c.pileggi@auckland.ac.nz (C.A.P.); j.markworth@auckland.ac.nz (J.F.M.); 2 School of Medicine, Deakin University, 75 Pigdons Road, Warun Ponds, VIC 3216, Australia; paul.lewandowski@deakin.edu.au

**Keywords:** ageing, endotoxaemia, high fat meals, inflammation, oxidative stress

## Abstract

Postprandial inflammation and endotoxaemia are determinants of cardiovascular and metabolic disease risk which are amplified by high fat meals. We aimed to examine the determinants of postprandial inflammation and endotoxaemia in older and younger adults following a high fat mixed meal. In a randomised cross-over trial, healthy participants aged 20–25 and 60–75 years (*n* = 15/group) consumed a high-fat breakfast and a low-fat breakfast. Plasma taken at baseline and post-meal for 5 h was analysed for circulating endotoxin, cytokines (monocyte chemotactic protein-1 (MCP-1), interleukin (IL)-1β, IL-6, and tumour necrosis factor-alpha (TNF-α)), lipopolysaccharide binding protein (LBP), and inflammatory gene expression in peripheral blood mononuclear cells (PBMC). Older subjects had lower baseline PBMC expression of glutathione peroxidase 1 (GPX-1) but greater insulin-like growth factor-binding protein 3 (IGFBP3) and circulating MCP-1 compared to younger subjects. After either meal, there were no age differences in plasma, chylomicron endotoxin, or plasma LBP concentrations, nor in inflammatory cytokine gene and protein expression (MCP-1, IL-1β, and TNF-α). Unlike younger participants, the older group had decreased superoxide dismutase (SOD)-2 expression after the meals. After a high-fat meal, older adults have no increased inflammatory or endotoxin response, but an altered oxidative stress gene response compared with younger adults. Healthy older adults, without apparent metabolic dysfunction, have a comparable postprandial inflammatory and endotoxaemia response to younger adults.

## 1. Introduction

Ageing is associated with dysfunction of the maintenance of cardiovascular and metabolic health, likely aggravated by the current western lifestyle resulting in declining health and increase chronic disease risk. Chronic low-grade inflammation is associated with ageing and contributes to morbidity and mortality [1]. The immunosenescence associated with ageing [2] manifests as an elevated basal immune status [3] along with insufficient immune activation after a challenge [4]. Evidence is mounting in support of chronic low-grade inflammation as a factor responsible in the development of insulin resistance and type 2 diabetes mellitus (T2DM) [5], along with other chronic illnesses such as cardiovascular disease or cancers [6] and, overall, as detrimental to healthy ageing [7]. Moreover, oxidative stress and inflammation contribute directly to the cellular senescence that helps perpetuate the condition of immune decline in ageing [8,9]. Age-related differences in monocyte expression of genes has begun to establish a characteristic senescent profile in the elderly [10,11]. Genes, such as insulin-like growth factor-binding protein 3 (IGFBP3) [10], cluster of differentiation (CD) 40 ligand CD40LG, and BCL2 agonist/killer 1 (BAK1) [11], are involved in cellular processes’ regulative cellular function, integrity, and responsiveness, and age-related changes in expression may contribute to differences in immune responsiveness in ageing.

Acute inflammatory responses can be initiated by meals, particularly those high in fat. The mechanisms of these postprandial inflammatory responses are highly reliant on the lipaemia caused by increased chylomicron formation and triacylglyceride (TAG) content in circulation. Chylomicron and TAG adherence to [12], and activation of, monocytes [13] provokes an acute immune response. Additionally, bacterial translocation across the gut, facilitated by fat absorption and chylomicron formation, triggers an immune response, manifesting as postprandial endotoxaemia [14]. The corresponding postprandial inflammatory state may further contribute to a systemic low-grade inflammation [15], already typically prevalent in ageing populations [9].

These postprandial lipaemic and inflammatory responses are already documented as elevated in metabolically-compromised adults, such as those displaying insulin resistance or T2DM [16,17,18,19], conditions found more prevalently in older adults [20,21]. Postprandial endotoxaemia is reliant on the formation of triacylglyceride-rich lipoproteins (TRL) [22,23,24], is evidently elevated in T2DM subjects [25], and is associated with increased intestinal permeability [26]. Ageing has sometimes been associated with digestive differences [27,28], changes to the gut microbiota [29], or gut barrier function [30] which could, therefore, contribute to elevated postprandial endotoxaemia after a high fat meal [31,32]. Furthermore, the exaggerated lipaemic response to high fat meals seen in older adults [33,34,35,36,37,38,39], including prolonged appearance [37,39] and overproduction of TRL, would likely contribute to greater postprandial endotoxaemia and inflammation in this population. Despite this evidence, studies examining the postprandial inflammatory responses of healthy older adults have yet to be conducted. Therefore, the aim of this study was to investigate the postprandial inflammatory response to a high fat mixed meal in older adults compared to younger adults. To achieve this, postprandial bacterial translocation was evaluated through measures of circulating endotoxin, acute phase protein responses, and monocyte gene expression of pyrogen specific receptors. Additional pathways of postprandial inflammatory activation were assessed through monocyte lipoprotein receptor gene expression. The resultant regulation and production of cytokines and antioxidants were examined, along with the senescent profile of monocytes. It was hypothesised that older adults would have greater postprandial endotoxaemia and inflammation, characterised by greater TRL transport of endotoxin and an exaggerated immune and oxidative stress response.

## 2. Materials and Methods

### 2.1. Subject Selection

**Figure 1:**
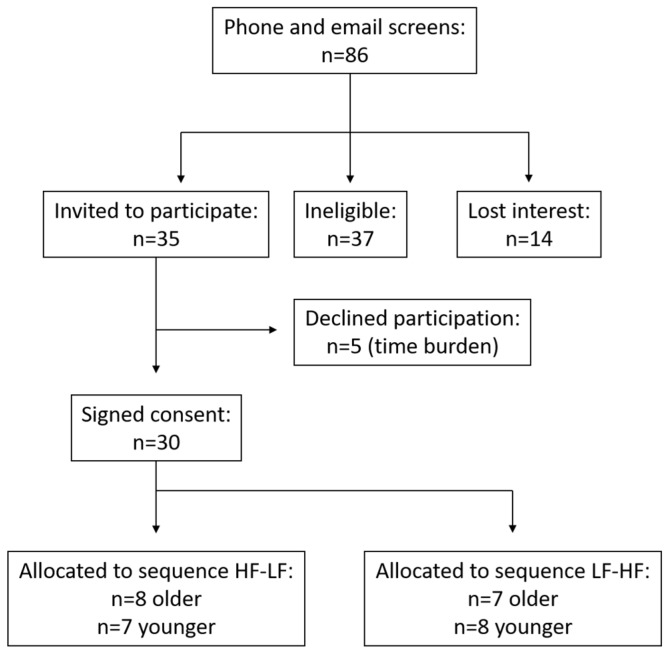
Participant eligibility, enrolment, and randomisation. HF, the high-fat meal; LF, the low fat meal.

Thirty healthy, community-dwelling subjects (*n* = 7 young females, *n* = 8 young males, *n* = 9 older females, *n* = 6 older males) from the Auckland region were recruited through local newspaper advertising to participate between October 2012 and July 2013. Eligible subjects were required to have a body mass index (BMI) between 18 and 30 kg/m^2^ and be between the ages of 20–25 years and 60–75 years. The original protocol to recruit older adults 70–75 was amended during recruitment due to difficulty identifying subjects meeting the eligibility requirements. Individuals with a history of cardiovascular or metabolic disease/conditions, or who used medications that may interfere with study endpoints (i.e., anti-inflammatory drugs, statin drugs) were not eligible for participation. Participant screening, enrolment, and randomisation are depicted in Figure 1.

This study was conducted according to the guidelines laid down by the Declaration of Helsinki and all procedures involving human subjects were approved by the University of Auckland Human Participants and Ethics Committee (Ref #8026). Written informed consent was obtained from all subjects. This study was registered with the Australian New Zealand Clinical Trials Registry (ID: ACTRN12612000515897).

### 2.2. Study Design and Treatments

In a randomised cross-over design, subjects received two test meals in a random sequence and served as his/her own control. Sequences were randomly generated using www.random.org [40] stratified by age group and allocated by concealed envelopes prior to the first visit. The primary outcome of elevated postprandial lipaemia in the elderly has been presented elsewhere [41]. Inflammatory cytokine gene expression, circulating endotoxin, cytokines, and markers of oxidative stress were assessed as secondary outcomes.

The high-fat meal (HF) was chosen as a standard test meal with a high-fat and protein load used previously to induce a postprandial inflammatory response [42] and was purchased from McDonald’s Restaurants in Auckland. The low fat meal (LF) was protein- and carbohydrate-matched to the HF meal based on standardly available nutritional information (Table 1) and was designed to follow the Australian Guide to Healthy Eating, while maintaining a low-fat load. Subjects were not blinded to meal identities.

### 2.3. Study Procedures

Subjects were asked to abstain from vigorous physical activity, high-fat foods, and anti-inflammatory medications and supplements the day prior to their visit to the Paykel Clinical Trial Unit at the Liggins Institute. Subjects arrived fasted on two separate occasions a minimum of 14 days apart. Anthropometric data were collected before a catheter was inserted into an antecubital vein and a baseline sample (time 0) was taken followed by consumption of the test breakfast. Blood samples were collected hourly for 5 h post-meal in blood collection tubes (Becton Dickinson, Franklin Lakes, NJ, USA) for serum and ethylenediaminetetraacetic acid (EDTA) plasma, and processed as described previously [43]. Plasma supernatants were processed under laminar flow using pyrogen-free consumables. Plasma for chylomicron separation was kept at 4 °C and processed within 6 h and the remaining plasma was collected in pyrogen-free microtubes and stored at −20 °C.

### 2.4. Chylomicron Isolation

Chylomicron separation was performed using gamma irradiated 4.7 mL OptiSeal tubes (Beckman Coulter, Brea, CA, USA) in an Optima™ MAX-XP model ultracentrifuge using a TLA-110 rotor, as described previously [44]. Density gradient solutions were prepared with 0.005% EDTA using pyrogen-free water according to Naito [45] and separation protocols were based on those of Kupke and Wörz-Zeugner [46]. Chylomicrons were separated by overlaying 3.5 mL plasma with 1.2 mL saline solution (density = 1.006 g/mL) and centrifuging at 117,000× *g* for 10 min. The visible chylomicron top layer was aspirated into pyrogen-free microtubes and corrected to a final collection volume of 1.4 mL using pyrogen-free saline solution to provide a standardised dilution factor. Chylomicron fractions were stored at −80 °C.

### 2.5. PBMC Isolation and RNA Extraction

Whole blood (2 mL), collected from EDTA blood collection tubes (Becton Dickinson, Franklin Lakes, NJ, USA) at 0, 2, and 4 h, was layered over 2 mL of Histopaque solution (Sigma-Aldrich, St. Louis, MO, USA) and centrifuged for 30 min at 400× *g* at room temperature. PBMCs were removed from the interface, washed twice with phosphate buffered saline (PBS), and RNA was extracted (Purelink RNA Mini Kit, Life Technologies, Waltham, MA, USA). DNA contamination was eliminated by DNase digestion from the cell lysate prior to RNA isolation. RNA concentration was measured using a NanoDrop (ND-1000 Spectrophotometer, Thermo Scientific, Waltham, MA, USA). RNA was reverse transcribed using a High Capacity RNA-to-cDNA Kit (Life Technologies, Waltham, MA, USA) as per the manufacturer’s instructions.

### 2.6. Quantitative Real-Time Reverse-Transcriptase Polymerase Chain Reaction (qPCR) Analysis

qPCR was performed using a LightCycler 480 (Roche Applied Science, Penzberg, Germany) using SYBR™ Green I DNA-binding dye. The geometric mean of human β-Actin, glyceraldehyde 3-phosphate dehydrogenase (GAPDH), and RNA18S genes was used as the endogenous control. Primers (Table S1) were obtained from Invitrogen (Life Technologies, Waltham, MA, USA). Samples were run in duplicate 10 μL reaction volumes with 1.25 ng cDNA per reaction. Results are expressed as absolute gene expression using the 2^−ΔCp^ method [47].

### 2.7. Biochemical Analysis

Biochemical measures of baseline plasma total cholesterol, low density lipoproteins (LDL) and high density lipoproteins (HDL), and postprandial triacylglycerides, glucose, and serum C-reactive protein (CRP) were carried out using a Hitachi 902 autoanalyser (Hitachi High Technologies Corporation, Tokyo, Japan) by enzymatic colorimetric assay (Roche, Mannheim, Germany). Postprandial plasma insulin was measured using an Abbott AxSYM system (Abbott Laboratories, Abbott Park, IL, USA) by microparticle enzyme immunoassay. The acute phase protein, plasma lipopolysaccharide binding protein (LBP), was assessed using a commercially available ELISA kit for quantification of human LBP (Abnova, Jhongli, Taiwan) at 0, 1, 2, and 3 h only, based on the postprandial responses reported by others [42]. Plasma inflammatory markers (*n* = 7 younger, *n* = 6 older), including tumour necrosis factor-alpha (TNF-α), monocyte chemotactic protein-1 (MCP-1), interleukin-1β (IL-β), and interleukin-6 (IL-6), were analysed using a flow cytometric multiplex array (Milliplex MAP Kit Human Cytokine Magnetic Bead Panel Assay, Millipore, Billerica, MA, USA) after the HF breakfast only, at 0, 2, and 4 h time points only matching with postprandial RNA analysis time points. Similarly, total superoxide dismutase (SOD) activity and total antioxidant status (TAS) were measured after the HF breakfast only, at 0, 2, and 4 h time points only, matching with postprandial RNA analysis time points, using commercial assay kits as per the manufacturer’s instructions (Sapphire Bioscience, Melbourne, Australia).

### 2.8. Endotoxin Analysis

Plasma and chylomicron endotoxin concentrations were determined (*n* = 12/group) using the Kinetic-QCL chromogenic Limulus Amebocyte Lysate (LAL) assay (Lonza, Cleveland, TN, USA) as per the manufacturer’s instructions. Chylomicron samples were diluted 1:100 with LAL reagent water (LRW) and heat inactivated at 70 °C for 10 min. Plasma samples were acid treated to remove inhibitory plasma proteins as described by Ketchum and Novitsky [48] in reagents prepared in LRW. In brief, samples were treated with 1.32 N nitric acid, heat treated at 37 °C for 5 min and centrifuged at 1500× *g* for 5 min. The supernatant was removed and neutralised with 0.55 N NaOH before dilution to 1:100 with LRW. The endotoxin concentration was expressed as endotoxin units (EU)/mL.

### 2.9. Statistical Analyses

The homeostatic model assessment of insulin resistance (HOMA-IR) was calculated from fasting glucose and insulin concentrations using the equation from Matthews et al. [49]. Maximum peak times (T_max_) were identified as the nominal sampling time corresponding with peak concentration. Sample size calculations have been described elsewhere for a primary outcome of postprandial lipaemia [41]; these were estimated to detect with 80% power a between-subject TAG incremental AUC (iAUC) difference of 4.77 mmol/L per h [35] with a significance level of *p* ≤ 0.05. Statistical analyses were conducted with SPSS (IBM, Armonk, NY, USA, version 21). Data are presented as means ± standard error of the mean (SEM), except T_max_ which is presented as median and interquartile range (IQR). Baseline concentrations were compared using two-way analysis of variance (ANOVA, treatment compared within-subject and age compared between subject). T_max_ was compared using a generalised estimation equation (GEE) with SPSS. The link identity was cumulative probit and an unstructured covariance matrix (UN); for binary variables (meal and age) a probit model was used. Protein expression of CRP, cytokines, and antioxidants were compared using two-factor repeated-measures ANOVA (time compared within-subject and age compared between subject), while three-factor repeated-measures ANOVA was used for all other analyses (treatment and time each compared within-subject and age compared between subject). Sidak post hoc tests were used for all multiple comparisons between groups. Where Mauchly’s sphericity test failed, the Huynh-Feldt correction was applied. Alpha was set at *p* < 0.05. Figures were generated with GraphPad Prism (GraphPad Software Inc., La Jolla, CA, USA, version 6.01).

## 3. Results

### 3.1. Subject Characteristics

There were no age differences for fasting measurements of BMI, plasma glucose, insulin, triglycerides, or HOMA-IR. Older subjects had higher fasting total cholesterol, LDL and HDL (*p* < 0.001, *p* = 0.008, and *p* < 0.001, respectively; Table 2). Differences in postprandial lipaemia are reported elsewhere [41]. In brief, older participants showed exaggerated and prolonged postprandial elevations of TAG. There were no differences in TAG iAUC between older and younger subjects after either meal, but iAUC was lower after the LF meal in both groups (treatment effect *p* < 0.05). However, the time to maximum TAG concentration (T_max_) was greater for older adults compared to younger adults after both the HF and LF meals (T_max_ HF 3 (IQR 3–4) vs. 2 (IQR 1.5–3) h and T_max_ LF 4 (IQR 3–5) vs. 3 (IQR 2–3) h for older vs. younger subjects, respectively; age effect *p* < 0.05).

### 3.2. Postprandial Endotoxaemia

**Figure 2:**
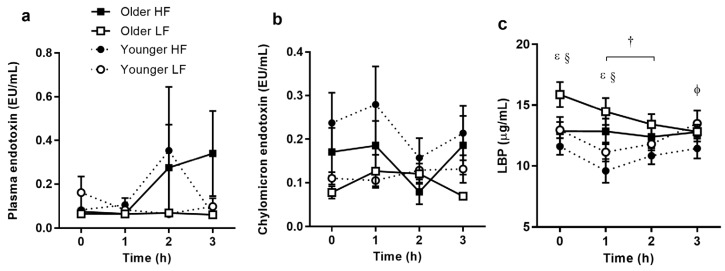
Endotoxin and lipopolysaccharide binding protein (LBP) responses to high-fat (HF) and low-fat (LF) breakfasts in older and younger subjects. Older high-fat (*filled squares*), older low-fat (*open squares*), younger high-fat (*filled circles*), younger low-fat (*open circles*). Values represent mean ± SEM in EU/mL for plasma endotoxin ((**a**); *n* = 12/group) and chylomicron endotoxin ((**b**); *n* = 12/group) and in μg/mL for LBP ((**c**); *n* = 15/group). There were no differences in plasma or chylomicron endotoxin responses between older and younger subjects. There were significant differences in the LBP response over time, dependent on age and treatment (age × time × treatment interaction of *p* < 0.05, three-factor repeated-measures ANOVA). ^ɛ^ *p* < 0.05 age difference after LF; ^§^ *p* < 0.05 treatment difference in older subjects; ^ϕ^ *p* < 0.05 treatment difference in younger subjects; ^†^ *p* < 0.05 time difference after LF in older subjects (Sidak corrected post hoc tests).

Plasma endotoxin concentration did not differ in the postprandial period (Figure 2a). Postprandial endotoxaemia tended to be greater after the HF breakfast (*p* = 0.156), with no evident impact of age (*p* = 0.84). Although endotoxaemia did not differ with age, older subjects tended to have prolonged endotoxaemia after 3 h (*p* = 0.059) while the younger group did not. Chylomicron endotoxin concentration tended to differ between breakfast treatments (*p* = 0.061) and treatment differences tended to depend on time (*p* = 0.097; Figure 2b). Chylomicron endotoxaemia after the HF breakfast varied more greatly between subjects, and post hoc analysis revealed a tendency towards greater differences at baseline between study visits, and at 1 and 4 h after the meal (*p* = 0.064, *p* = 0.052, and *p* = 0.133, respectively). Changes in LBP concentration differed between treatments and age groups (*p* = 0.013, Figure 2c). Older subjects had greater LBP concentrations at baseline and 1 h during the LF challenge than during the HF breakfast (*p* = 0.008 and 0.036, respectively). This corresponded with higher LBP concentrations in the older participants at baseline and 1 h during the LF challenge (*p* = 0.017 and *p* = 0.024 respectively). With the higher baseline concentration in older participants before the LF meal, the older group experienced a significant decrease in LBP concentration by 2 h post-meal (baseline vs. 2 h; *p* = 0.016). The younger group had higher concentrations of LBP at 3 h after the LF than after the HF breakfast.

### 3.3. PBMC Senescence RNA Expression

**Figure 3:**
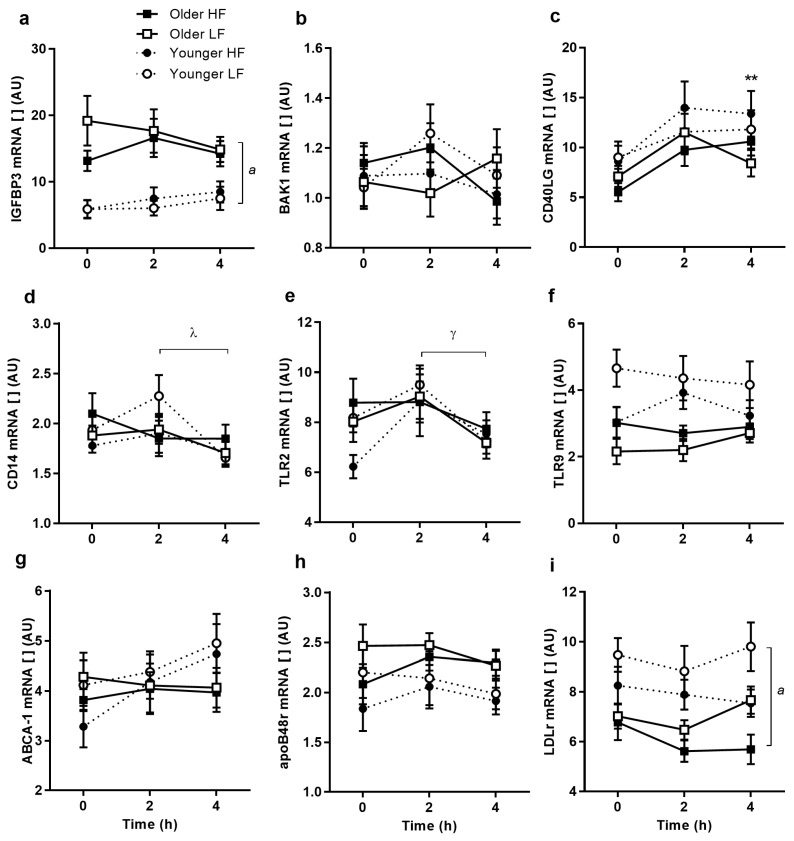
Relative senescence-related, endotoxaemia-related and lipid receptor peripheral blood mononuclear cells (PBMC) gene expression responses to high-fat (HF) and low-fat (LF) breakfasts in older and younger subjects. Older high-fat (*filled squares*), older low-fat (*open squares*), younger high-fat (*filled circles*), younger low-fat (*open circles*). Values represent mean ± SEM (*n* = 15/group) in arbitrary units (AU) for IGFBP3 (**a**), BAK1 (**b**), CD40LG (**c**), CD14 (**d**), TLR2 (**e**), TLR9 (**f**), ABCA-1 (**g**), ApoB48r (**h**), and LDLr (**i**), respectively. There were no differences in BAK1 TLR2, TLR9, ABCA-1, or ApoB48r responses between older and younger adults. There were significant age differences in LDLr gene expression (age effect of *p* < 0.05) and changes over time for CD40LG and TLR2 (time effect of *p* < 0.05 and *p* < 0.01, respectively). There were significant age differences and time differences, dependent on treatment in the IGFBP3 response (time × treatment interaction of *p* < 0.01, age effect of *p* < 0.01, three-factor repeated-measures ANOVA). CD14 expression was significantly different between older and younger adults over time (time × age interaction of *p* < 0.05). *^a^ p* < 0.05 main age difference; ** *p* < 0.01 change from baseline; ^λ^ *p* < 0.05 time difference in younger subjects; ^γ^ *p* < 0.01 time difference (Sidak corrected post hoc tests).

IGFBP3 expression was greater in the older group (*p* = 0.001) and tended to be higher before the LF meal (*p* = 0.073) causing a significant time-treatment interaction (*p* = 0.005; Figure 3a). BAK1 expression was no different between groups and did not differ with feeding (Figure 3b). Both groups had increased CD40LG expression at 4 h after both meals (*p* = 0.002; Figure 3c).

### 3.4. PBMC RNA Expression of Endotoxaemic Activation

Younger subjects had decreased CD14 expression at 4 h compared to 2 h (*p* = 0.005; Figure 3d), while older subjects showed no changes after meal ingestion. Toll-like receptor (TLR) 2 (TLR2) response was no different between groups after either meal but was higher at 2 h than 4 h (*p* = 0.002; Figure 3e). TLR9 expression tended to be lower overall in older participants (*p* = 0.055; Figure 3f).

### 3.5. PBMC RNA Expression of Activation by Lipoproteins

No change in ATP-binding cassette transporter 1 (ABCA-1) expression was observed after either meal in either age group (Figure 3g). Apolipoprotein B48 receptor (ApoB48r) expression tended to be higher in older participants (*p* = 0.111), higher after the LF meal (*p* = 0.087) and higher at baseline (*p* = 0.15; Figure 3h). The older group had lower LDL receptor (LDLr) expression (*p* = 0.038; Figure 3i).

### 3.6. PBMC Inflammatory Cytokine Gene Expression

**Figure 4:**
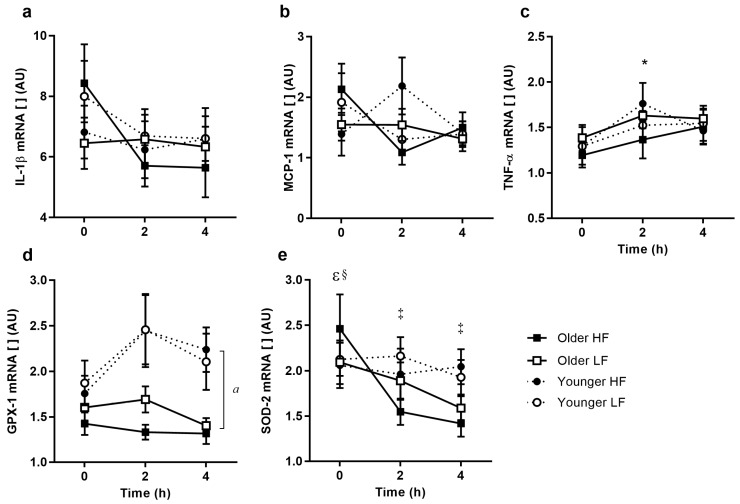
Relative inflammatory cytokine and oxidative stress peripheral blood mononuclear cells (PBMC) gene expression responses to high-fat (HF) and low-fat (LF) breakfasts in older and younger subjects. Older high-fat (*filled squares*), older low-fat (*open squares*), younger high-fat (*filled circles*), and younger low-fat (*open circles*). Values represent mean ± SEM (*n* = 15/group) in arbitrary units (AU) for IL-1β (**a**), MCP-1 (**b**), TNF-α (**c**), GPX-1 (**d**), and SOD-2 (**e**), respectively. There were no differences in IL-1β, MCP-1, or TNF-α, responses between older and younger subjects. There were significant age differences in GPX-1 gene expression (age effect of *p* < 0.05 each respectively) and changes over time for IL-1β and TNF-α (time effect of *p* < 0.05 each respectively). The SOD-2 response differed over time, dependent on age and treatment (age × time × treatment interaction of *p* < 0.05, three-factor repeated-measures ANOVA). *^a^ p* < 0.05 main age difference; * *p* < 0.05 change from baseline; ^ɛ^ *p* < 0.05 age difference after LF; ^§^ *p* < 0.05 treatment difference in older subjects; ^‡^ *p* < 0.05 time difference after HF in older subjects (Sidak corrected post hoc tests).

IL-1β expression changed after meal ingestion (*p* = 0.03) and tended to decrease compared to baseline (*p* = 0.107). MCP-1 expression did not change after meal ingestion and the response was similar between age groups (Figure 4a,b). TNF-α expression increased over time (*p* = 0.033; Figure 4c), but was not different between breakfasts or age groups.

### 3.7. PBMC RNA Expression of Oxidative Stress Response

Younger subjects had higher glutathione peroxidase 1 (GPX-1) expression (*p* = 0.014; Figure 4d), however, this did not change after either meal. SOD-2 expression decreased at 2 and 4 h after meal ingestion in the older group, but not the younger group (*p* < 0.001; Figure 4e). For this reason, older subjects had lower SOD-2 expression at 2 and 4 h compared to younger subjects (*p* = 0.044 and *p* = 0.004, respectively).

### 3.8. Cytokine and Antioxidant Protein Expression

**Figure 5:**
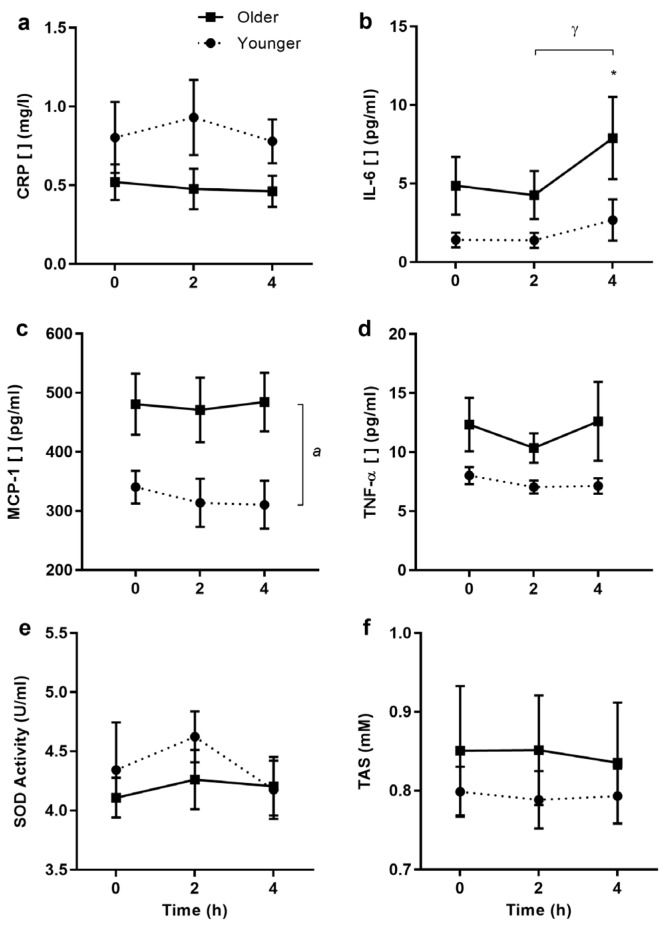
Circulating C-reactive protein, cytokine protein concentrations, and antioxidant status in older and younger subjects after the high-fat breakfast only. C-reactive protein (CRP), superoxide dismutase activity (SOD), and total antioxidant status (TAS, *n* = 15/group), and cytokine protein concentrations in older (*filled squares*, *n* = 7) and younger adults (*filled circles*, *n* = 6), respectively. Values represent mean ± SEM for CRP (**a**) in mg/L, IL-6 (**b**) in pg/mL, MCP-1 (**c**) in pg/mL, TNF-α (**d**) in pg/mL, SOD (**e**) in U/mL, and TAS (**f**) in mM. There were no differences in CRP, IL-6, TNF-α, SOD, and TAS responses between older and younger subjects. There were significant age differences in MCP-1 protein concentrations (age effect of *p* < 0.05) and changes over time for IL-6 (time effect of *p* < 0.01, two-factor repeated-measures ANOVA). *^a^ p* < 0.05 main age difference; * *p* < 0.05 change from baseline; ^γ^ *p* < 0.01 time difference (Sidak corrected post hoc tests).

There were no differences between age groups in postprandial concentrations of serum CRP (*p* = 0.84, Figure 5a), and plasma IL-1β (*p* = 0.436), IL-6, and TNF-α (Figure 5b,d; *p* = 0.111 and *p* = 0.112 respectively), although IL-6 and TNF-α tended to be higher in older participants. Cytokine concentrations measured for IL-1β and IL-6 were very low with reference to the standard curve, and IL-6 (but not IL-1β) increased between baseline and 4 h in both age groups (*p* = 0.007). IL-1β did not change after the meal for older or younger subjects (*p* = 0.336 time effect; iAUC −87.9 ± 90.0 (older subjects) vs. −19.8 ± 12.7 (younger subjects); *p* = 0.504). MCP-1 concentration was higher in the older group (*p* = 0.028, Figure 5c), and unchanged with meal ingestion. SOD activity and TAS were not different between age groups nor affected by HF meal ingestion (Figure 5d,e).

## 4. Discussion

This study demonstrates that in an otherwise healthy older cohort, postprandial inflammation is not exaggerated compared to younger adults. Our results indicate that baseline differences in immune status may be present in healthy older adults, but that any such differences are not associated with differences in the postprandial immune response to a high fat meal. The similarities in immune response between older and younger adults are in spite of exaggerated postprandial lipaemia in these older subjects [41] and suggest that the inflammatory response to a high-fat meal is not exacerbated in older age despite age-related differences in the basal immune profile.

Previous studies have shown that a high-fat meal induces postprandial inflammation; however, the magnitude and the specific measurable markers of this inflammation varies between studies [50], and are reliant on the precise meal components included [51,52,53,54], as well as the health status of subjects [16]. Inflammatory gene expression [16], plasma cytokines [18], and endotoxaemia [25] are reported to be particularly elevated after high-fat or high-caloric meals [55] in metabolically-compromised adults, such as those with T2DM and metabolic syndrome. These acute meal responses may contribute to a state of chronic low-grade inflammation, accelerating the development of insulin resistance and cardiovascular disease through progression of atherosclerosis [56,57]. To our surprise, no marked difference in postprandial endotoxaemia or inflammation was seen between younger and older subjects in the current study, suggesting that older adults do not display a greater acute inflammatory response to ingestion of a single high-fat meal.

Postprandial inflammation is elevated after a high fat meal in part due to monocyte activation by circulating lipoproteins resulting from postprandial lipaemia [58]. Although older adults have increased numbers of circulating TRLs [41], we found that this did not correspond with higher postprandial gene expression of monocyte lipoprotein receptors, apoB48r and LDLr, suggesting no greater postprandial potential for monocyte activation by lipoproteins in older adults. Metabolically, greater insulin resistance is associated with greater postprandial endotoxaemia [25,59]; as HOMA-IR was not different between older and younger participants, this supports the idea that the two conditions may be linked, and may explain the absence of postprandial endotoxaemia in these older subjects. Our results suggest that despite the higher incidence of insulin resistance or hyperlipidaemia typical in older adults, and the greater postprandial inflammatory effects of these conditions [16,25], older individuals free from metabolic dysfunction do not experience greater postprandial inflammation.

Along with metabolic effects on postprandial inflammation, gut barrier function may have a crucial role in regulating the immune response to a high fat meal. Postprandial endotoxaemia is increased after a high-fat meal [60] corresponding with elevated lipaemia [24,42], a finding recently linked to direct chylomicron translocation of endotoxin [14,22,23,42]. Although the absorptive and immune function of the gut has been suggested to be impaired with ageing [61,62], our finding of equal postprandial plasma and chylomicron endotoxaemia between older and younger adults contradicts this notion, and supports the concept that ageing itself does not conclusively impair gut barrier function [27,28]. Furthermore, this suggests that older adults in the current study had maintained gut barrier function in spite of exaggerated chylomicronaemia.

The postprandial immune response to endotoxin is initiated by acute LBP production [42], which also facilitates the endotoxaemic immune response by activation of NF-κB through CD14 and TLR4, while other bacterial components act through TLR2 [63] and TLR9. Corresponding with no postprandial age differences in endotoxaemia, we saw no greater postprandial LBP concentration or induction of PBMC genes involved in bacterial immune defence in older participants after a high-fat meal. Furthermore, the typical [64] basal elevation of LBP in older subjects did not contribute to a greater endotoxaemic response, indicating that an elevated immune status in these individuals does not increase the magnitude of postprandial endotoxaemia. Similarly, postprandial cytokine genes and protein expression were no different between age groups, indicating no deficit or elevation in postprandial immune response associated with ageing.

Oxidative stress is also suggested to contribute to the postprandial inflammatory response through cytokine production [65,66]. Although IL-6 concentrations were elevated after meal ingestion, this change was equal between age groups. Despite the age similarity in cytokine response, older participants had lower SOD-2 gene expression which decreased after the high fat meal while younger subjects maintained expression levels equal to baseline, similar to other reports [67]. However, while the age differences in gene expression may suggest an inadequate postprandial antioxidant response in older adults, protein antioxidant expression of SOD activity and TAS were no different between age groups. As absolute protein responses do not appear to be compromised in these older subjects, compensatory mechanisms to maintain adequate antioxidant responsiveness may be at play in the healthy elderly. Increased postprandial GPX-1 expression has been reported following a meal high in saturated fat as a response to increased reactive oxygen species production [68]. As we also reported lower baseline GPX-1 expression that was maintained after the ingestion of either meal, these older subjects may have lower antioxidant capacity, which suggests that basal immune status may contribute to postprandial antioxidant capacity but not necessarily immune response in older individuals.

While the lack of elevated immune response to a high-fat meal in older participants likely indicates no impairment of acute activation, we investigated the possibility that immunosenescence could also elicit such a moderate acute immune responsiveness, particularly since oxidative stress has been associated with cellular immunosenescence [8,9]. Analysis of expression of genes associated with immunosenescence, such as impaired apoptosis regulation (BAK1) and cellular immune activation (CD40LG) [11], indicated no obvious impairment of the immune response in these older subjects. The elevated expression of IGFBP3 that we observed is typical of the senescence profile reported in older adults [10], and suggests that, despite characteristic cellular senescent changes in these older adults, postprandial immune activation remains unaffected.

## 5. Conclusions

Our results highlight that, despite typically elevated postprandial lipaemia, otherwise metabolically-healthy older adults do not have an exaggerated postprandial immune or endotoxaemic response to a standard high-fat mixed meal. These findings provide the first description of the comparative evaluation of postprandial immune responses in older adults. These findings indicate that the basal health and metabolic status of older adults should be considered as a confounding factor in the study of postprandial immune responses in an ageing population, since these healthy elderly show no impairment of postprandial response. This suggests that the maintenance of health status into older age may be protective against the detrimental effects of acute high fat meal ingestion, and that older adults are at no greater risk than younger adults for chronic low-grade inflammation initiated by fat ingestion.

## Figures and Tables

**Table 1:** Macronutrient composition of high- and low-fat breakfasts.

Item Name	Weight (g)	Macronutrients (g) ^1^			Energy (kcal)
		Carbohydrates	Fat	Protein	
**High fat breakfast**					
Sausage and Egg Muffin Sandwich (×2)	162	25.2	21	23.4	390
Hash Brown (×2)	56	13.5	10.1	1.5	150
Total		77.4	62.2	49.8	1080
**Low fat breakfast**					
Rolled Oats	37	20.8	1.9	5.0	120
1% Cottage Cheese	167	4.5	1.0	19.7	110
Mixed Grain Bread	42	11.2	2.2	5.1	90
Reduced Fat Peanut Butter, Smooth	25	8.4	9.4	4.4	140
Fresh Peach	154	14.6	0.3	1.4	60
Trim Milk	365	17.9	1.8	14.2	150
Total		77.4	16.6	49.8	670

^1^ Values presented are based on nutrient panel data obtained from the website of the fast food restaurant and from packaging of the low fat breakfast items.

**Table 2:** Baseline subject characteristics.

Measure ^1^	Unit	Younger Subjects (*n* = 15) ^2^	Older Subjects (*n* = 15) ^2,3^
Age	years	22.7 ± 0.4	67.3 ± 1.5 ***
BMI	kg/m^2^	23.7 ± 0.8	24.4 ± 1.0
Glucose	mmol/L	5.1 ± 0.1	5.2 ± 0.1
HOMA-IR		2.1 ± 0.2	1.9 ± 0.2
Cholesterol	mmol/L	4.0 ± 0.1	5.0 ± 0.1 ***
LDL	mmol/L	2.5 ± 0.1	3.0 ± 0.1 **
HDL	mmol/L	1.3 ± 0.0	1.8 ± 0.1 ***
TAG	mmol/L	0.8 ± 0.0	0.9 ± 0.0
Insulin	μU/mL	9.2 ± 0.8	8.7 ± 1.2

^1^ BMI: body mass index; HOMA-IR: homeostatic model assessment of insulin resistance; LDL: low density lipoproteins; HDL: high density lipoproteins; TAG: triacylglyceride; ^2^ Values presented as means ± standard error of the mean (SEM) over both treatments; ^3^ Main effects and interactions were analysed by two-factor repeated-measures analysis of variance (ANOVA; treatment and age). There were no differences between group baseline values between treatment days; *** *p* < 0.001, ** *p* < 0.01 compared with younger subjects.

## Supplementary Materials

The following are available online at http://www.mdpi.com/2072-6643/9/4/354/s1, Table S1: Primer sequences for targeted peripheral blood mononuclear cell (PBMC) gene expression.

## References

[1] Shaw A.C., Goldstein D.R., Montgomery R.R. (2013). Age-dependent dysregulation of innate immunity. Nat. Rev. Immunol..

[2] Franceschi C., Bonafe M., Valensin S., Olivieri F., De Luca M., Ottaviani E., De Benedictis G. (2000). Inflamm-aging. An evolutionary perspective on immunosenescence. Ann. N. Y. Acad. Sci..

[3] Krabbe K.S., Pedersen M., Bruunsgaard H. (2004). Inflammatory mediators in the elderly. Exp. Gerontol..

[4] Bruunsgaard H., Pedersen A.N., Schroll M., Skinhøj P., Pedersen B.K. (1999). Impaired production of proinflammatory cytokines in response to lipopolysaccharide (LPS) stimulation in elderly humans. Clin. Exp. Immunol..

[5] Duncan B.B., Schmidt M.I., Pankow J.S., Ballantyne C.M., Couper D., Vigo A., Hoogeveen R., Folsom A.R., Heiss G. (2003). Low-grade systemic inflammation and the development of type 2 diabetes: The atherosclerosis risk in communities study. Diabetes.

[6] Wagner M., Samdal Steinskog E.S., Wiig H. (2015). Adipose tissue macrophages: The inflammatory link between obesity and cancer?. Expert Opin. Ther. Targets.

[7] Akbaraly T.N., Hamer M., Ferrie J.E., Lowe G., Batty G.D., Hagger-Johnson G., Singh-Manoux A., Shipley M.J., Kivimäki M. (2013). Chronic inflammation as a determinant of future aging phenotypes. Can. Med. Assoc. J..

[8] Cannizzo E.S., Clement C.C., Sahu R., Follo C., Santambrogio L. (2011). Oxidative stress, inflamm-aging and immunosenescence. J. Proteom..

[9] Singh T., Newman A.B. (2011). Inflammatory markers in population studies of aging. Ageing Res. Rev..

[10] Hong M.G., Myers A.J., Magnusson P.K.E., Prince J.A. (2008). Transcriptome-wide assessment of human brain and lymphocyte senescence. PLoS ONE.

[11] Abdul Rahman A., Abdul Karim N., Abdul Hamid N.A., Harun R., Wan Ngah W.Z. (2013). Senescence-related changes in gene expression of peripheral blood mononuclear cells from octo/nonagenarians compared to their offspring. Oxid. Med. Cell. Longev..

[12] Candore G., Colonna-Romano G., Balistreri C.R., Di Carlo D., Grimaldi M.P., Listì F., Nuzzo D., Vasto S., Lio D., Caruso C. (2006). Biology of longevity: Role of the innate immune system. Rejuvenation Res..

[13] Klop B., Proctor S.D., Mamo J.C., Botham K.M., Cabezas M.C. (2012). Understanding postprandial inflammation and its relationship to lifestyle behaviour and metabolic diseases. Int. J. Vasc. Med..

[14] Laugerette F., Vors C., Peretti N., Michalski M.-C. (2011). Complex links between dietary lipids, endogenous endotoxins and metabolic inflammation. Biochimie.

[15] Calder P.C., Ahluwalia N., Brouns F., Buetler T., Clement K., Cunningham K., Esposito K., Jönsson L.S., Kolb H., Lansink M. (2011). Dietary factors and low-grade inflammation in relation to overweight and obesity. Br. J. Nutr..

[16] Van Dijk S., Mensink M., Esser D., Feskens E., Muller M., Afman L.A. (2012). Responses to high-fat challenges varying in fat type in subjects with different metabolic risk phenotypes: A randomized trial. PLoS ONE.

[17] Ceriello A. (2006). Effects of macronutrient excess and composition on oxidative stress: Relevance to diabetes and cardiovascular disease. Curr. Atheroscler Rep..

[18] Manning P.J., Sutherland W.H.F., McGrath M.M., De Jong S.A., Walker R.J., Williams M.J.A. (2008). Postprandial cytokine concentrations and meal composition in obese and lean women. Obesity.

[19] Patel C., Ghanim H., Ravishankar S., Chang L.S., Viswanathan P., Mohanty P., Dandona P. (2007). Prolonged reactive oxygen species generation and nuclear factor-κb activation after a high-fat, high-carbohydrate meal in the obese. J. Clin. Endocrinol. Metab..

[20] Stolk R.P., Pols H.A.P., Lamberts S.W.J., De Jong P.T.V.M., Hofman A., Grobbee D.E. (1997). Diabetes mellitus, impaired glucose tolerance, and hyperinsulinemia in an elderly population: The rotterdam study. Am. J. Epidemiol..

[21] Marques-Vidal P., Mazoyer E., Bongard V., Gourdy P., Ruidavets J.B., Drouet L., Ferrières J. (2002). Prevalence of insulin resistance syndrome in southwestern france and its relationship with inflammatory and hemostatic markers. Diabetes Care.

[22] Laugerette F., Vors C., Géloën A., Chauvin M.-A., Soulage C., Lambert-Porcheron S., Peretti N., Alligier M., Burcelin R., Laville M. (2011). Emulsified lipids increase endotoxemia: Possible role in early postprandial low-grade inflammation. J. Nutr. Biochem..

[23] Levels J.H., Abraham P.R., van den Ende A., van Deventer S.J. (2001). Distribution and kinetics of lipoprotein-bound endotoxin. Infect. Immun..

[24] Deopurkar R., Ghanim H., Friedman J., Abuaysheh S., Sia C.L., Mohanty P., Viswanathan P., Chaudhuri A., Dandona P. (2010). Differential effects of cream, glucose, and orange juice on inflammation, endotoxin, and the expression of toll-like receptor-4 and suppressor of cytokine signaling-3. Diabetes Care.

[25] Harte A.L., Varma M.C., Tripathi G., McGee K.C., Al-Daghri N.M., Al-Attas O.S., Sabico S., O’Hare J.P., Ceriello A., Saravanan P. (2012). High fat intake leads to acute postprandial exposure to circulating endotoxin in type 2 diabetic subjects. Diabetes Care.

[26] Mani V., Hollis J.H., Gabler N.K. (2013). Dietary oil composition differentially modulates intestinal endotoxin transport and postprandial endotoxemia. Nutr. Metab..

[27] Grassi M., Petraccia L., Mennuni G., Fontana M., Scarno A., Sabetta S., Fraioli A. (2011). Changes, functional disorders, and diseases in the gastrointestinal tract of elderly. Nutr. Hosp..

[28] Woudstra T., Thomson A.B.R. (2002). Nutrient absorption and intestinal adaptation with ageing. Best Pract. Res. Clin. Gastroenterol..

[29] Saffrey M.J. (2014). Aging of the mammalian gastrointestinal tract: A complex organ system. Age.

[30] Russell R.M. (1992). Changes in gastrointestinal function attributed to aging. Am. J. Clin. Nutr..

[31] Cani P.D., Bibiloni R., Knauf C., Waget A., Neyrinck A.M., Delzenne N.M., Burcelin R. (2008). Changes in gut microbiota control metabolic endotoxemia-induced inflammation in high-fat diet-induced obesity and diabetes in mice. Diabetes.

[32] Pendyala S., Walker J.M., Holt P.R. (2012). A high-fat diet is associated with endotoxemia that originates from the gut. Gastroenterology.

[33] Cohn J.S., McNamara J.R., Cohn S.D., Ordovas J.M., Schaefer E.J. (1988). Postprandial plasma lipoprotein changes in human subjects of different ages. J. Lipid Res..

[34] Puga G.M., Meyer C., Mandarino L.J., Katsanos C.S. (2013). Increased plasma availability of l-arginine in the postprandial period decreases the postprandial lipemia in older adults. Nutrition.

[35] Puga G.M., Meyer C., Everman S., Mandarino L.J., Katsanos C.S. (2011). Postprandial lipemia in the elderly involves increased incorporation of ingested fat in plasma free fatty acids and small (S_f_ 20-400) triglyceride-rich lipoproteins. Am. J. Physiol. Endocrinol. Metab..

[36] Borel P., Mekki N., Boirie Y., Partier A., Grolier P., Alexandre-Gouabau M.C., Beaufrere B., Armand M., Lairon D., Azais-Braesco V. (1997). Postprandial chylomicron and plasma vitamin e responses in healthy older subjects compared with younger ones. Eur. J. Clin. Investig..

[37] Krasinski S.D., Cohn J.S., Schaefer E.J., Russell R.M. (1990). Postprandial plasma retinyl ester response is greater in older subjects compared with younger subjects: Evidence for delayed plasma clearance of intestinal lipoproteins. J. Clin. Investig..

[38] Borel P., Mekki N., Boirie Y., Partier A., Alexandre-Gouabau M.C., Grolier P., Beaufrere B., Portugal H., Lairon D., Azais-Braesco V. (1998). Comparison of the postprandial plasma vitamin a response in young and older adults. J. Gerontol. A Biol. Sci. Med. Sci..

[39] Cardinault N., Tyssandier V., Grolier P., Winklhofer-Roob B.M., Ribalta J., Bouteloup-Demange C., Rock E., Borel P. (2003). Comparison of the postprandial chylomicron carotenoid responses in young and older subjects. Eur. J. Nutr..

[40] Haahr M. Random integer set generator. www.random.org/integer-sets/.

[41] Milan A.M., Nuora A., Pundir S., Pileggi C.A., Markworth J.F., Linderborg K.M., Cameron-Smith D. (2016). Older adults have an altered chylomicron response to a high-fat meal. Br. J. Nutr..

[42] Ghanim H., Abuaysheh S., Sia C.L., Korzeniewski K., Chaudhuri A., Fernandez-Real J.M., Dandona P. (2009). Increase in plasma endotoxin concentrations and the expression of toll-like receptors and suppressor of cytokine signaling-3 in mononuclear cells after a high-fat, high-carbohydrate meal: Implications for insulin resistance. Diabetes Care.

[43] Milan A.M., D’Souza R.F., Pundir S., Pileggi C.A., Barnett M.P.G., Markworth J.F., Cameron-Smith D., Mitchell C. (2015). Older adults have delayed amino acid absorption after a high protein mixed breakfast meal. J. Nutr. Health Aging.

[44] Oikawa S., Mizunuma Y., Iwasaki Y., Tharwat M. (2010). Changes of very low-density lipoprotein concentration in hepatic blood from cows with fasting-induced hepatic lipidosis. Can. J. Vet. Res..

[45] Naito H.K. (1986). Lipoprotein Separations Using the TL-100 Tabletop Ultrafuge.

[46] Kupke I.R., Wörz-Zeugner S. (1986). Sequential microultracentrifugation of lipoproteins in 100ul of serum. J. Lipid Res..

[47] Livak K.J., Schmittgen T.D. (2001). Analysis of relative gene expression data using real-time quantitative PCR and the 2-δδCT method. Methods.

[48] Ketchum P.A., Novitsky T.J., Thomas J.E. (2000). Assay of endotoxin by limulus amebocyte lysate. Septic Shock Methods and Protocols.

[49] Matthews D., Hosker J., Rudenski A., Naylor B., Treacher D., Turner R. (1985). Homeostasis model assessment: Insulin resistance and b-cell function from fasting plasma glucose and insulin concentrations in man. Diabetologia.

[50] Jackson K.G., Poppitt S.D., Minihane A.M. (2012). Postprandial lipemia and cardiovascular disease risk: Interrelationships between dietary, physiological and genetic determinants. Atherosclerosis.

[51] Raz O., Steinvil A., Berliner S., Tovit R., Justo D., Shapira I. (2013). The effect of two iso-caloric meals containing equal amounts of fats with a different fat composition on the inflammatory and metabolic markers in apparently healthy volunteers. J. Inflamm..

[52] Camargo A., Peña-Orihuela P., Rangel-Zúñiga O.A., Pérez-Martínez P., Delgado-Lista J., Cruz-Teno C., Marín C., Tinahones F., Malagón M.M., Roche H.M. (2014). Peripheral blood mononuclear cells as in vivo model for dietary intervention induced systemic oxidative stress. Food Chem. Toxicol..

[53] Ghanim H., Sia C.L., Upadhyay M., Korzeniewski K., Viswanathan P., Abuaysheh S., Mohanty P., Dandona P. (2010). Orange juice neutralizes the proinflammatory effect of a high-fat, high-carbohydrate meal and prevents endotoxin increase and toll-like receptor expression. Am. J. Clin. Nutr..

[54] Burton-Freeman B., Talbot J., Park E., Krishnankutty S., Edirisinghe I. (2012). Protective activity of processed tomato products on postprandial oxidation and inflammation: A clinical trial in healthy weight men and women. Mol. Nutr. Food Res..

[55] Schwander F., Kopf-Bolanz K.A., Buri C., Portmann R., Egger L., Chollet M., McTernan P.G., Piya M.K., Gijs M.A., Vionnet N. (2014). A dose-response strategy reveals differences between normal-weight and obese men in their metabolic and inflammatory responses to a high-fat meal. J. Nutr..

[56] De Vries M.A., Klop B., Janssen H.W., Njo T.L., Westerman E.M., Castro Cabezas M. (2014). Postprandial inflammation: Targeting glucose and lipids. Adv. Exp. Med. Biol..

[57] De Vries M.A., Klop B., Eskes S.A., van der Loos T.L.J.M., Klessens-Godfroy F.J.M., Wiebolt J., Janssen H.W., Westerman E.M., Castro Cabezas M. (2014). The postprandial situation as a pro-inflammatory condition. Clin. Investig. Arterioscler..

[58] Alipour A., Van Oostrom A.J.H.H.M., Izraeljan A., Verseyden C., Collins J.M., Frayn K.N., Plokker T.W.M., Elte J.W.F., Cabezas M.C. (2008). Leukocyte activation by triglyceride-rich lipoproteins. Atertioscler. Thromb. Vasc. Biol..

[59] Creely S.J., McTernan P.G., Kusminski C.M., Fisher F.M., Da Silva N., Khanolkar M., Evans M., Harte A.L., Kumar S. (2007). Lipopolysaccharide activates an innate immune system response in human adipose tissue in obesity and type 2 diabetes. Am. J. Physiol. Endocrinol. Metab..

[60] Erridge C., Attina T., Spickett C.M., Webb D.J. (2007). A high-fat meal induces low-grade endotoxemia: Evidence of a novel mechanism of postprandial inflammation. Am. J. Clin. Nutr..

[61] Katz D., Hollander D., Said H.M., Dadufalza V. (1987). Aging-associated increase in intestinal permeability to polyethylene glycol 900. Dig. Dis. Sci..

[62] Tran L., Greenwood-Van Meerveld B. (2013). Age-associated remodeling of the intestinal epithelial barrier. J. Gerontol. A Biol. Sci. Med. Sci..

[63] Schröder N.W.J., Morath S., Alexander C., Hamann L., Hartung T., Zähringer U., Göbel U.B., Weber J.R., Schumann R.R. (2003). Lipoteichoic acid (LTA) of streptococcus pneumoniae and staphylococcus aureus activates immune cells via toll-like receptor (TLR)-2, lipopolysaccharide-binding protein (LBP), and CD14, whereas TLR-4 and MD-2 are not involved. J. Biol. Chem..

[64] Gonzalez-Quintela A., Alonso M., Campos J., Vizcaino L., Loidi L., Gude F. (2013). Determinants of serum concentrations of lipopolysaccharide-binding protein (LBP) in the adult population: The role of obesity. PLoS ONE.

[65] Liu L., Zhao S.P., Wen T., Zhou H.N., Hu M., Li J.X. (2008). Postprandial hypertriglyceridemia associated with inflammatory response and procoagulant state after a high-fat meal in hypertensive patients. Coron. Artery Dis..

[66] Devaraj S., Wang-Polagruto J., Polagruto J., Keen C.L., Jialal I. (2008). High-fat, energy-dense, fast-food-style breakfast results in an increase in oxidative stress in metabolic syndrome. Metabolism.

[67] Ghanim H., Sia C.L., Korzeniewski K., Lohano T., Abuaysheh S., Marumganti A., Chaudhuri A., Dandona P. (2011). A resveratrol and polyphenol preparation suppresses oxidative and inflammatory stress response to a high-fat, high-carbohydrate meal. J. Clin. Endocrinol. Metab..

[68] Yubero-Serrano E.M., Gonzalez-Guardia L., Rangel-Zuñiga O., Delgado-Casado N., Delgado-Lista J., Perez-Martinez P., Garcia-Rios A., Caballero J., Marin C., Gutierrez-Mariscal F.M. (2013). Postprandial antioxidant gene expression is modified by mediterranean diet supplemented with coenzyme q10 in elderly men and women. Age.

