# Supplementary material for: Comparisons of the Postprandial Inflammatory and Endotoxaemic Responses to Mixed Meals in Young and Older Individuals: A Randomised Trial
Source: Nutrients. 2017 Apr 2;9(4):354. doi: 10.3390/nu9040354 (PMC5409693; doi:10.3390/nu9040354)
Supplement: Supplementary file 1 [file nutrients-09-00354-s001.docx]

Supplementary Materials: Comparisons of the Postprandial Inflammatory and Endotoxaemic Responses to Mixed Meals in Young and Older Individuals: A Randomised Trial

Amber M Milan, Shikha Pundir, Chantal A Pileggi, James F Markworth, Paul A Lewandowski, David Cameron-Smith

**Table S1**. Primer sequences for targeted peripheral blood mononuclear cell (PBMC) gene expression.

| Primer set | Forward | Reverse |
| --- | --- | --- |
| β-Actin | TGGCACCCAGCACAATGAA | CTAAGTCATAGTCCGCCTAGAAGCA |
| GAPDH | GGCAGTGAAAGGGACGATTCT | GCTGCTTTAACATAGGCCAGGT |
| RNA18S | GATGGTAGTCGCCGTGCC | GCCTGCTGCCTTCCTTGG |
| ABCA1 | GGCGCTTTGCTCCTTGTTTT | CCGTGGCTGGTCATTAACTGT |
| apoB48r | GCCAGTCACCTCTTCCTCTG | AGGATGCACAGACTGGCTCT |
| BAK1 | CGGCAGAGAATGCCTATGAG | AGTCAGGCCATGCTGGTAGA |
| CD14 | AGAGGTTCGGAAGACTTATCG | TCGGAGAAGTTGCAGACGC |
| CD40LG | AATCCTCAAATTGCGGCACA | TTAACGGTCAGCTGTTTCCCA |
| GPX-1 | TATCGAGAATGTGGCGTCCC | TCTTGGCGTTCTCCTGATGC |
| IGFBP3 | TCAACGCTAGTGCCGTCAG | AATGGAGGGGGTGGAACTTG |
| IL-1β | TTCGAGGCACAAGGCACAA | TGGCTGCTTCAGACACTTGAG |
| LDLr | ACCACAGAGGATGAGGTCCA | TGACCATCTGTCTCGAGGGG |
| MCP-1 | GCAATCAATGCCCCAGTCAC | CTTGAAGATCACAGCTTCTTTGGG |
| SOD-2 | GGCCTACGTGAACAACCTGA | TGGGCTGTAACATCTCCCTTG |
| TLR2 | ATCCTCCAATCAGGCTTCTCT | ACACCTCTGTAGGTCACTGTTG |
| TNF-α | AGCCCATGTTGTAGCAAACC | TGAGGTACAGGCCCTCTGAT |
